# Psoriasis, COVID‐19 and shielding

**DOI:** 10.1111/bjd.20055

**Published:** 2021-07-01

**Authors:** G. Becher, A.D. Burden

**Affiliations:** West Glasgow Ambulatory Care Hospital Glasgow UK; Institute of Infection, Immunity and InflammationUniversity of Glasgow UK

## Abstract

**Linked Article:** Mahil *et al. Br J Dermatol* 2021; **185**:80–90.

The COVID‐19 pandemic has presented clinicians managing immune‐mediated inflammatory diseases (IMIDs) such as psoriasis, with many challenges. Patients with severe psoriasis have an increased prevalence of risk factors for severe COVID‐19 including obesity, hypertension, diabetes and male sex.^1^ Moreover, many systemic treatments for psoriasis are known to increase the risk of severe infection. Therefore, it is understandable that in the early stages of the pandemic, patients on conventional targeted systemic therapies were considered to be at higher risk of severe COVID‐19 infection. In addition to risk‐mitigating behaviours such as social distancing recommended by the World Health Organization, those who were thought to be more vulnerable, for instance those on immunosuppressants, were advised to adopt stricter measures of social isolation including distancing themselves from other members of their household.^2^, ^3^ There was a pressing need to establish whether patients on immunosuppressive or immunomodulatory medications should continue on their medications.

By early 2020, international registries for patients with IMIDs, such as PsoProtect and SECURE‐AD Registry, had been established for healthcare professionals to record cases of COVID‐19 and the impact of systemic treatments on outcomes of the infection. Registry data have shown that patients on targeted systemic treatment such as biologics do not appear to be at increased risk of severe COVID‐19 compared with those on standard systemic agents or no treatment.^4^ Counterintuitively, patients receiving treatments that block cytokines such as tumour necrosis factor and interleukin‐17A appear to have a lower rate of hospitalization from COVID‐19.^5^ This could be due to immunomodulatory effects of these drugs on the overproduction of cytokines that contribute to deleterious consequences of severe COVID‐19 infection. An alternative explanation is behavioural – perhaps patients on biologics are less likely to be exposed to the virus because of particularly effective shielding.

PsoProtectMe and the CORE‐UK study (COVID‐19 Rheumatology Register) are online self‐reporting registries for patients with psoriasis and rheumatoid arthritis, respectively, to record their experiences and behaviour during this pandemic. In this issue of the *BJD*, Mahil *et al*. characterize the shielding behaviours in 3720 participants with IMIDs from 74 countries, and report on how these differ by treatment type.^6^ Patients were divided into three treatment groups: standard systemic agents (e.g. methotrexate), targeted systemic treatments (biologics and Janus kinase inhibitors) and no systemic treatment. Of the 3720 participants, 2262 reported shielding behaviour. Of those shielding, 1632 were based in the UK and, interestingly, only 899 of them reported having been specifically advised to shield. Along with male sex, obesity and comorbidity burden, the investigators found that the use of targeted systemic treatment was most strongly associated with increased shielding behaviour, compared with standard systemic therapy or no therapy. The reasons for this are unclear, and further studies would be useful to ascertain if there are differences in patient perception of risk across different treatment groups and why this might be. Shielding correlated positively with anxiety and depression, and inversely with larger households and cigarette smoking, suggesting individual factors contribute, as well as the advice received.

Despite the limitations of online self‐reporting, for instance access to and literacy in information technology, as well as recall bias, this study provides valuable information both for clinicians caring for patients with IMIDs during this pandemic, and for public health agencies responsible for advising patients on risk‐mitigating behaviour. Registries such as PsoprotectMe are vital in helping clinicians understand more about the complicated relationships between psoriasis, its treatments, patient behaviours and COVID‐19 infection.

## Author Contribution

**Gabrielle Louise Becher:** Writing‐original draft (lead); Writing‐review & editing (equal). **David Burden:** Writing‐original draft (supporting); Writing‐review & editing (equal).

## References

[bjd20055-bib-0001] Gelfand JM, Armstrong AW, Stacie Bell S *et al*. National Psoriasis Foundation COVID‐19 Task Force guidance for management of psoriatic disease during the pandemic: Version 1. J Am Acad Dermatol 2020; 83:1704–16. 32891785 10.1016/j.jaad.2020.09.001 PMC7471802

[bjd20055-bib-0002] Guidance on shielding and protecting people who are clinically extremely vulnerable from COVID‐19. February 2021. Department of Health & Social Care and Public Health England. Available at: https://www.gov.uk/government/publications/guidance‐on‐shielding‐and‐protecting‐extremely‐vulnerable‐persons‐from‐covid‐19/guidance‐on‐shielding‐and‐protecting‐extremely‐vulnerable‐persons‐from‐covid‐19 (last accessed 10 March 2021).

[bjd20055-bib-0003] World Health Organization. Information note on COVID‐19 and NCDs. 23 March 2020. Available at: https://www.who.int/publications/m/item/covid‐19‐and‐ncds (last accessed 10 March 2021).

[bjd20055-bib-0004] Gianfrancesco M, Hyrich KL, Al‐Adely S *et al*. Characteristics associated with hospitalisation for COVID‐19 in people with rheumatic disease: data from the COVID‐19 Global Rheumatology Alliance physician‐reported registry. Ann Rheum Dis 2020; 79:859–66. 32471903 10.1136/annrheumdis-2020-217871 PMC7299648

[bjd20055-bib-0005] Mahil SK, Dand N, Mason KJ *et al*. Factors associated with adverse COVID‐19 outcomes in patients with psoriasis – insights from a global registry‐based study. J Allergy Clin Immunol 2020; 147:60–71. 33075408 10.1016/j.jaci.2020.10.007 PMC7566694

[bjd20055-bib-0006] Mahil SK, Yates M, Langan S *et al*. Risk‐mitigating behaviours in people with inflammatory skin and joint disease during the COVID‐19 pandemic differ by treatment type: a cross‐sectional patient survey. Br J Dermatol 2021; 10.1111/bjd.19755 PMC9214088 33368145

